# Tumor reversion: a dream or a reality

**DOI:** 10.1186/s40364-021-00280-1

**Published:** 2021-05-06

**Authors:** Avantika Tripathi, Anjali Kashyap, Greesham Tripathi, Joni Yadav, Rakhi Bibban, Nikita Aggarwal, Kulbhushan Thakur, Arun Chhokar, Mohit Jadli, Ashok Kumar Sah, Yeshvandra Verma, Hatem Zayed, Amjad Husain, Alok Chandra Bharti, Manoj Kumar Kashyap

**Affiliations:** 1 Amity Stem Cell Institute, Amity Medical School, Amity University Haryana, Panchgaon, Haryana, Manesar (Gurugram) -122413 India; 2 Department of Biotechnology, Thapar Institute of Engineering & Technology, Patiala, Punjab India; 3 Department of Zoology, Molecular Oncology Laboratory, University of Delhi (North Campus), New Delhi, 110007 India; 4 Department of Medical Laboratory Technology, Amity Medical School, Amity University Haryana, Panchgaon, Haryana, Manesar (Gurugram) India; 5 Department of Pathology and Laboratory Medicine, Medanta-The Medicity, Haryana, Gurugram India; 6 Department of Toxicology, C C S University, Meerut, UP 250004 India; 7 Department of Biomedical Sciences, College of Health Sciences, QU Health, Qatar University, Doha, Qatar; 8 Centre for Science & Society, Indian Institute of Science Education and Research (IISER), Bhopal, India; 9 Innovation and Incubation Centre for Entrepreneurship (IICE), Indian Institute of Science Education and Research (IISER), Bhopal, India

**Keywords:** Tumor reversion, TCTP1, SIAH1, Phenotype reversion, Revertant, PTMs

## Abstract

Reversion of tumor to a normal differentiated cell once considered a dream is now at the brink of becoming a reality. Different layers of molecules/events such as microRNAs, transcription factors, alternative RNA splicing, post-transcriptional, post-translational modifications, availability of proteomics, genomics editing tools, and chemical biology approaches gave hope to manipulation of cancer cells reversion to a normal cell phenotype as evidences are subtle but definitive. Regardless of the advancement, there is a long way to go, as customized techniques are required to be fine-tuned with precision to attain more insights into tumor reversion. Tumor regression models using available genome-editing methods, followed by in vitro and in vivo proteomics profiling techniques show early evidence. This review summarizes tumor reversion developments, present issues, and unaddressed challenges that remained in the uncharted territory to modulate cellular machinery for tumor reversion towards therapeutic purposes successfully. Ongoing research reaffirms the potential promises of understanding the mechanism of tumor reversion and required refinement that is warranted in vitro and in vivo models of tumor reversion, and the potential translation of these into cancer therapy. Furthermore, therapeutic compounds were reported to induce phenotypic changes in cancer cells into normal cells, which will contribute in understanding the mechanism of tumor reversion. Altogether, the efforts collectively suggest that tumor reversion will likely reveal a new wave of therapeutic discoveries that will significantly impact clinical practice in cancer therapy.

**Supplementary Information:**

The online version contains supplementary material available at 10.1186/s40364-021-00280-1.

## Introduction

Cancer is a complex genetic disease that can be either solid or hematological type. The GLOBOCAN report in 2018 estimated that the total number of new cases and death is predicted to be 18.1 and 9.6 million, respectively [1]. Uncontrolled proliferation and loss of cellular and molecular architecture are typical characteristics of cancers [2]. For many years, the somatic mutation theory (SMT) was used as the basis for explaining the cause behind carcinogenesis. SMT mostly relates to non-inheritance cancers, including 90–95% of all cancer types. In 1914, Boveri was the first person to introduce the SMT first explanation, which showed that for changing the cell’s phenotype, the genotype had to be changed [3]. Over time, it has been claimed that a single somatic cell contains multiple DNA mutations in cancer, indicating that cancers are monoclonal [4]. Their central premise was (1) cancer is a defect of the control of cell proliferation, and (2) quiescent state is the default state for metazoan cells [5]. Later, another theory came into the picture called “The tissue organization field theory of carcinogenesis” (TOFT), which considers DNA mutations not the cause of cancer, as in SMT, but as the effect [6]. Towards the end of the nineteenth century, Bold, Cohnheim, and Ribbert provided theory based on the interactions between tissues; cancer produced in embryonic residues and epithelial cells do not contain any special proliferative power, but that their proliferation results from being freed from the restrictions imposed by normal tissue organization. Their basic premises are: **(I)** Motility and proliferation are the default cellular states, **(II)** Carcinogenesis, and neoplasia are the outcome of the tissue architecture defects [7]. In the initial phase, carcinogens will disrupt the parenchyma’s normal interactions and an organ’s stroma. It appears as the primary target (The morphological field of developing organisms). Neoplastic cells are reprogrammed to work as normal cells in “normal tissue”^.^. Carcinogens initially disrupt normal interactions among parenchymal and stromal cells of an organ (an equivalent of the “morphogenetic fields” of a growing organism). An emergent (supracellular) phenomenon is involved in the cause of Carcinogenesis and neoplasia. According to TOFT, pro-carcinogenic agents disrupt and interfere with the normal tissue architecture, and lead to the destruction of cell-to-cell signaling and conciliate the genomic integrity [8].

A large number of switches such as chromosomal instability, loss of heterozygosity, accumulation of mutations, DNA methylation, and intron retention (particularly in TSGs), escape of the immunosurveillance of the immune system, aberrations in metabolism, defect in DNA machinery, uncontrolled cell division, neo-angiogenesis, dysregulation of post-transcriptional modifications & post-translational modifications (PTMs), a nexus of the tumor microenvironment, and changes in the extracellular matrix composition, are collectively responsible for the transformation of normal cells into the malignant form [9].

The different output or so-called differentially-regulated molecules between cancers vs. normal cells/tissues have been used as potential anti-cancer therapy targets for different malignancies [10]. Targeting different types of tumors had been the center of attraction using other chemical probes, antibodies, or mimetic to see an impact on the tumor volume and or survival of the animals and the patients. In recent years, changing cancer cell’s profile into normal (also called phenotype reversion or tumor reversion) also received a lot of attention.

The process of tumor reversion was first described in the twentieth century while studying ovarian teratomas. Through embryonic differentiation, tumor cells could rise to normal cells [11]. Interestingly, SV40 or polyoma infected NIH/3T3 cells were enriched, and those possessed enhancements of improved contact inhibition, and sensitivity, along with attrition in the capacity to produce a tumor. These variants of the parental cell lines are described as “revertants”. The tissues of embryonic origin reverted cancerous cells into normal using the guidance cues from the normal microenvironment [2].

Reversion of tumor cells involves the regeneration of the whole or as a part of the standard growth control mechanisms, which disappeared in the malignant cells. Still, tumor progression has been a serious concern as it poses severe challenges to biomedical scientists and clinicians worldwide. Several therapeutic agents, including standard of care (SOC) drugs, have been used to treat cancer patients to inhibit/stop the tumor progression. In contrast to tumor progression, the phenomenon of tumor reversion is less studied. Tumor reversion is a biological process involving reprogramming of tumor cells that overcome the aberrancies such as loss of heterozygosity, mutations, inactivation of TSGs, hyperactivation of oncogenes, and eventually leading to tumor phenotype conversion into normal. The in vitro and in vivo approaches are used to manipulate the cellular machinery for correcting the behavior of tumor cells in different malignancies [2].

Reports are showing that the morphogenetic fields can guide tissue to behave differently. When applied on tumor cells growing in the vicinity of normal tissues as embryonic through cellular reprogramming, it could revert into a normal phenotype [12]. Several studies successfully used molecular reprogramming for reverting the tumor phenotype [12–14]. Recently, similar experimental approaches applied to several different malignancies, including leukemia, [15] breast cancer, [15] prostate cancer, [16] ovarian cancer, [17] and liver cancer, [18] to study tumor reversion.

The high-throughput techniques such as DNA or oligonucleotide microarrays were used to identify differentially regulated genes between normal vs. cancerous tissues. This technology provided significant insights on significantly dysregulated genes and helped to understand the changes in an individual or a set of genes in different stages of the tumor.

Malignant cells originate from the normal cells, so the major challenge is to reminiscence the molecular pathophysiology behind tumor reversion, which has been overlooked largely due to limited researcher interest and involvement in this area of research. Still, a significant number of evidence, based on the observations from different studies showed that tumor microenvironment, post-transcriptional modifications, PTMs, chemical compounds including anti-sense oligonucleotides (ASOs), and miRs raise the possibility that further advancement in this field could make it possible to use tumor reversion as an alternative strategy or synergistically along with currently available SOCs.

In this review, we discussed current updates in the field of tumor reversion including currently available in vitro, in vivo, and 3D culture-based models to study tumor reversion, different molecular events involved, compounds exploited for tumor reversion, and above all the challenges along with critical scientific thoughts to implement multi-omics [19], and the current state-of-the-art technologies to delineate the molecular process of tumor reversion.

### Molecules involved in tumor reversion

**Fig. 1:**
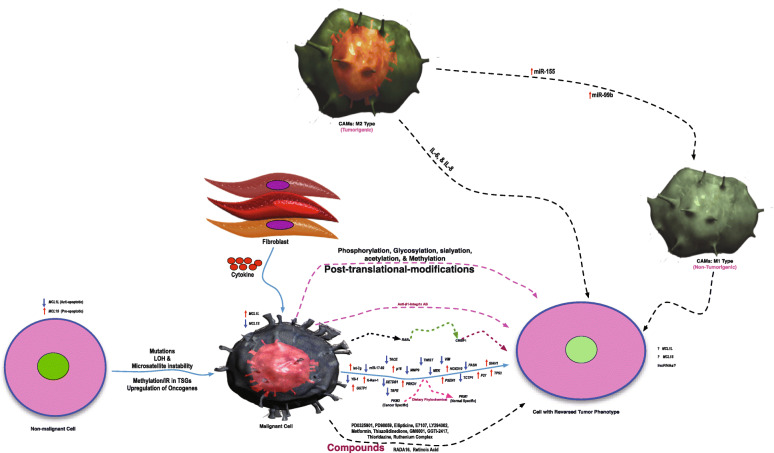
Different molecular alterations involved in tumor reversion. Molecular mechanism of tumor reversion involving different alternations including PTMs such as phosphorylation, glycosylation, and other molecular changes such as microRNAs, transcription factors, RNA splicing events, the impact of the tumor microenvironment, tumor-associated macrophages, and epigenetic modifications. The arrow with an upward direction (**↑**) denotes an increase in the expression, and the arrow with a downward direction (**↓**) denotes a decrease in the expression. In the figure, gene symbols in *italic* means denoting gene/mRNA, and *non-italic* means denoting protein

**Fig. 2:**
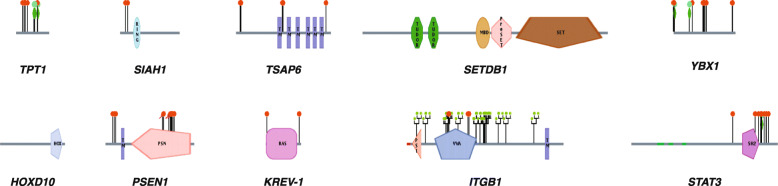
Protein Architecture of different proteins involved in the tumor reversion. Using the human protein reference database, the architecture of proteins involved in tumor reversion or phenotypic tumor reversion has been shown include TPT1, SIAH1, TSAP6, SETDB1, YBX1, HOXD10, PSEN1, KREV-1, ITGB1, and STAT3

Several molecular processes and molecules were reported to be involved in tumor reversion (Table 1, and Fig. 1); still, only a selected group of researchers focused on dissecting the complicated biological process of tumor reversion globally. The protein architecture of different molecules involved in tumor reversion is provided in Fig. 2. Some of these critical molecules involved in tumor reversion are:

**Table 1:** List of molecules directly or indirectly involved in process of tumor reversion (phenotype from tumor to normal) in human and mouse cancer cell lines/models

Name of Molecule	Gene Symbol	Experimental Settings	Type of Malignancy	Findings of the Study	Relevant Reference
Translationally controlled tumor protein (TCTP)	*TPT1*	H1 parvovirus was used for preparing the revertants. TCTP was inhibited using anti-sense oligonucleotide or disruptive small RNA molecules	Breast (BT20, T47D, and MDA-MB-231) and leukemia (K562 and U937)	Reduction of TPT1/TCTP expression by anti-sense cDNA and siRNA in various breast cancer and leukemia cell lines was observed and the study concluded that TCTP gene downregulation is necessary for tumor reversion or to have a suppressed tumor phenotype.	Tuynder et al 2002 [15]
Seven in absentia homolog 1 (E3 ubiquitinprotein ligase)	*SIAH1*	H1 parvovirus was used for preparing the revertants.	Breast (BT20, T47D, and MDA-MB-231) and leukemia (K562 and U937)	*SIAH1* transfectants observed to have suppressive tumor phenotype as compared with the control.	Tuynder et al 2002, [15]
Presenilin 1	*PSEN1*	The anti-sense complementary to PSEN1 was stable transfected in U937 cells.	Leukemic (K562 & U937)	*TP53* and *P21* genes endorsed *PSEN1* expression. In an In vivo SCID mouse model, inhibition of PSEN1 led to suppression of tumor phenotype, retarded growth, and induction of apoptosis.	Roperch et al 1998, [20]
Signal transducer and activator of transcription 3	*STAT3 (Transcription Factor)*	Multiple myeloma cell line (RPMI8226) was infected with H1 parvovirus to make the revertant. The parental cells vs revertant cells compared using in vivo proteomics labeling technique SILAC followed by LCMS/MS analysis.	Multiple myeloma (RPMI8226)	The revertant cell line has a suppressed tumor characteristic compared to the parent cell line. Inhibition of STAT3 suppresses malignant phenotype, and induces apoptosis in vitro and in vivo state.	Ge et al 2010, [21]
K-rev-1 (RAP1A) GTPase	*KREV1*	Upon prolonged of PC3 cells with Azatyrosine, resistant clones were obtained and analyzed in both in vitro as well as in vivo conditions.	Prostate Cancer cell lines (TSU-Prl, DU-145, and PC-3)	The resistant PC3 clones showed very low number of colony forming ability, and complete loss of tumorigenicity observed in one clone. Further, KREV-1 expression was high in revertant as compared with parental cell line further confirmed induction of tumor reversion due to azatyrosine.	Benoit et al 1995, [16]
Rhoassociated protein kinase	*ROCK*	The inhibitors of ROCK as well as of mammalian target of rapamycin (mTOR) kinase inhibitors can substitute for all transcription factors (TFs) to be sufficient to reprogram breast cancer cells into progenitor cells.	Breast Cancer (MDA-MB-468, MDA-MB-231, and HCC2157)	In vitro and in vivo tumorigenesis tests have shown that induced fat-like cells lose proliferation and tumorigenicity. Reprogramming was possible by phenotypic changes by using ROCK–mTOR kinase inhibitors in breast cancer cell line that are induced progenitor-like cells (iPLs). These inhibitors prohibit locally the recurrence in mouse model.	Yuan et al 2018, [22]
Breast and ovarian cancer susceptibility protein 1	*BRCA1*	Targeted NGS was applied using circulating cell-free DNA (cfDNA) isolated from pre and postprogression plasma samples derived from high-grade ovarian carcinoma (HGOC) to profile BRCA mutations in rucaparib (PARP inhibitor)	Ovarian cancer	An important resistance mechanism to platinum-based chemotherapies and PARP inhibitors in *BRCA1*-mutant cancers was studied using cfDNA. This showed that the acquisition of *BRCA1* reversion mutations capable of restoring the protein function.	Lin et al 2019 [17]
SET Domain Bifurcated 1 (Transcription regulatory protein)	*SETDB1*	The gene regulatory network (GRNs) analysis in previously datasets led to identification of identify core TFs (CDX2, ELF3, HNF4G, PPARG, and VDR) that control the cellular state.	Colorectal cancer cell lines (Caco2, HCT116, SW480, and SW620)	RNAseq analysis of single cell, showed upregulation of SETDB1 expression in stem like cancerous cells as compare with normal cells, and an elimination of SETDB1 in Caco2 cells shows KRT20+ population. This depletion led to changes of stem cancer cell phenotype into post-mitotic cells and led to restorage of normal morphology in colon cancer patient derived organoid.	Lee et al 2020, [23]
*β1-integrin*	*TGB*	Tyrosine kinase inhibitor (Tyrphostin AG 1478), Specific MAPK inhibit (PD98059), and I3K inhibitor (LY294002) were use to tre t the brea t ca er cell line.	Breast cancers (MCF7, HS578T, and MDA-MB-231)	An overexpression of E-cadherin gave rise to partial reversion, but when these transfected cells supplemented with beta1 integrin, PI3K or MAPK inhibitors, complete tumor reversion was achieved in MDA-MB-231 & MCF7.	Wang et al 2002, [24]
Homeobox D10 (Transcription factor)	*HOXD10*	Manipulation of MDA-MB-231 (breast cancer cell line) to restore the HOXD10 expression using retroviral gene expression system in a three-dimensional laminar pattern (3DlrBM).	Breast Cancer (MDA-MB-231)	Restorations of HOXD10 expression led to decreased expression of A3 integrin and reduced cellular proliferation and the cells were able to form acinar structures polarized in nature. Additionally, HOXD10 expression led to inhibition of tumorigenesis induced by MDA-MB-231 in mouse xenografts.	Carrio et al 2005, [25]
Matrix Metalloprotein ase9	*MMP9*	An MMP9 inhibitor GM6001 and another inhibitor of clinical grade (Marimastat) were tested for cellular behavior in 3D culture.	Breast Cancer (S1 and T4–2 cells of the HMT3522)	Both the agents were able to induce tumor reversion as compared with the control in these cell lines as evident with the morphological changes.	Beliveau et al 2010, [26] Coussens et al 2002, [27]
Mitogenactivated protein kinase.	*MEK*	Different isogenic variants of MCF10A were treated using MEKi inhibitor PD032590.	Transformed variants (isogenic cell lines in MCF10A, a normal human breast epithelial cell line) with different tumorigenic potential were used.	The MEKi inhibitor reverted the surfaceome changes in MCF10A cell line. Among isogenic variants of MCF10A, the most sensitive to MEKi were MEK^DD^, BRAF^V600E^, and EGFR^L858R^	Leung et al 2020, [28]
YB-1 ( Transcription factor)	*YBX1*	Genome editing technique CRISPR/Cas9 was used to knockout.	Melanoma (MDA-MB-435), and MCF-7	Quashing of YBX1 led to inhibition of not only proliferation but also cell cycle arrest and apoptosis in melanoma as well as in breast cancer cells and generates inevitable cancer stem cell differentiation. This leads to reduce tumorigenic potential of CSCs in in vitro as well as in vivo conditions.	Yang et al 2019,[29]
Lipogenic enzyme fatty acid synthase (FASN)	*FASN*	Short hairpin RNA (shRNA) based inhibition of FASN	MCF10A progression series (MCF10A untransformed cells, MCF10AneoT and MCF10AT non-malignant cells, MCF10DCIS.com ductal carcinoma in situ cells and MCF10Ca1a, Ca1d and Ca1h malignant cell lines)	The Inhibition of FASN lead to suppression of lipogenesis in CA1d cells and also induced reversion of these cells into nonmalignant phenotype in breast cancer cells.	Gonzalez-Guerrico et al. 2016,[30]
Retinoic acid receptor α (RARα)	RARα	RARα agonist Am580 (4-[(5,6,7,8-tetrahydro-5,5,8,8-tetramethyl2-naphthyl)carboxamido]benzoic acid)	MMTV-Myc female mice fed on 0.3 mg/kg/day diet with RARα agonist	The RARα activation induce reexpression of CRBP1 which leads to reversion of the malignant phenotype.	Bosch et al 2012,[31]

#### Translationally controlled tumor protein 1 (TCTP1)

**Fig. 3:**
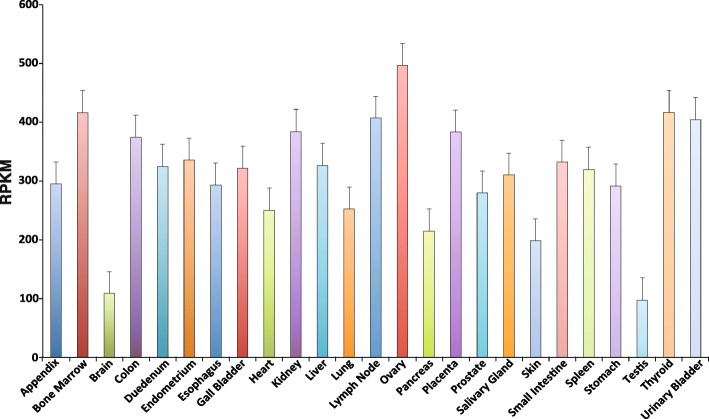
The mRNA expression of TPT1 across different normal human tissues. An mRNA expression of the *TPT1* gene has been shown across all possible normal human tissue samples including (from left-➔ to right) appendix, bone marrow, brain, colon, duodenum, endometrium, esophagus, gall bladder, heart, kidney, liver, lung, lymph node (LN), ovary, pancreas, placenta, prostate, salivary gland, skin, small intestine, spleen, stomach, testis, thyroid, and urinary bladder. The value of the expression is shown in form of Reads Per Kilobase of transcript per million mapped reads (RPKM), which are the normalized unit for denoting transcript expression

In humans, the Translationally Controlled Tumor Protein (TCTP) is encoded by the *TPT1* gene, which is located on 13q12-q1413. It consists of six exons and five introns [32]. TCTP is a secretory calcium-binding protein whose expression has been reported in biological fluids such as saliva and semen. *TPT1* is a direct target gene of *TP53*. The conditional KO mice of *TPT1* showed retardation in the development of the brain and leads to death in the perinatal stage. The expression of *TPT1* across different normal human samples has been shown in Fig. 3. The *TPT1* mRNA expression profile clearly shows that at least a minimum count of 97.21 RPKM was present across all the normal organs. The protein-protein interactions (PPIs) of TCTP proteins are provided in Supplementary Table-1.

PPIs can be identified using phage display, immunoprecipitation, and Yeast two-hybrid (Y2H) techniques. Over 200 PPIs involve TCTP protein; as evident from different PPI analysis platforms like Y2H, affinity captures mass spectrometry, Affinity Capture-Luminescence, or Affinity Capture-Western [33].

Y2H is a molecular technique that is used for the identification of PPIs especially the physical interactions [34, 35]. The functioning of the cellular machinery depends on the physical interactions between domains of several transcription factors. These domains are structurally and functionally different: DNA binding domain (BD), and DNA activation domain (AD). Among these, BD binds to the DNA sequence upstream of the reporter gene, and AD stimulates the reporter gene expression. The protein in questions (Query) fuses with BD and known as BAIT, the library of proteins fuse with AD and known as prey [36]. Y2H takes advantage of the fact that gene transcription requires the binding of two domains of a transcriptional activator protein. These domains are called the DNA-binding domain and the activator domain. For two-hybrid analysis, each domain is fused to one of two candidate interacting proteins. If these proteins interact then a functional transcriptional activator is formed. This triggers the transcription of a reporter gene, which gives an observable change in phenotype. In Y2H, a reporter expressed if there is an interaction between two proteins. In Y2H, one can screen for interacting partners without purifying the protein. There are some drawbacks of Y2H as well including the limitations of testing for pairwise interactions only. Y2H is prone to a high false-positive rate as well. Some of the critical PPIs were between *TCTP* and *MCL1*, *TCTP* and *SPP1*, & *TCTP* and *BCL2L1* as listed in Supplementary Table 1.

Dysregulation of *TCTP* in breast cancer led to the restructuring of the tumor and initiation of making duct-like structures giving the mnemonic impression of normal breast tissue [15]. Overexpression of the TCTP has been associated with a poor prognosis in ovarian cancer. The siRNA knockdown of the *TPT1* gene showed retarded growth of the ovarian carcinoma cell lines in vitro suggesting its role in cell proliferation [37].

#### SIAH E3 ubiquitin protein ligase 1 (*SIAH1)*

An E3 ubiquitin-protein ligase that is encoded by the gene *SIAH1*, [32] involved in ubiquitination and degradation of specific proteins via proteasome through an interaction of SIAH1 with NUMB. Mutations in SIAH1 inhibit the β-catenin degradation, and these mutations have been reported in gastric cancer samples as well [38]. Not only SIAH1 but also its homologs (Siah1 & Siah2) interacts with DCC (deleted in colorectal cancer) and subjects it to proteolysis through the ubiquitin-proteasome pathway [39]. Inactivation of SIAH1 is associated with hepatocellular carcinoma (HCC) tumor progression [40]. Overexpression of SIAH1 in colorectal cancer (CRC) led to the suppression of cellular proliferation and invasion of malignant cells. In contrast, SIAH1 knockdown promotes both proliferation as well as invasion of CRC cells [41]. Overexpression of SIAH1 in U937 cells not only induce apoptosis but also led to tumor reversion [20].

#### Presenilin (PSEN1)

Human presenilin protein encoded by the *PSEN1* gene, which is located on locus 14q24.3. This protein possesses one transmembrane domain, as well as one PSN domain [32]. PSEN1 is an important γ-secretase complex member that plays a crucial role in the NOTCH signaling pathway. An upregulation and over-amplification of the *PSEN1* were observed in cancerous tissues and cell lines of gastric origin. It was positively correlated with lymph node (LN) metastasis and poor survival rate in gastric cancer patients [42]. An ASO blocking *PSEN1* that was used in leukemic cell lines (K562 and U937) induces apoptosis, and reverts the tumor phenotype in the cell lines as well as in vivo in the SCID mouse model [20].

#### Tumor suppressor activated pathway 6 (TSAP6)

The official gene symbol for the tumor suppressor activated pathway 6 (TSAP6) is *STEAP3* that is mapped to 2q14.2. It is a cell cycle control protein, which possesses six transmembrane domains. Due to its active role in tumor reversion/suppression, it was given the name TSAP6 [10]. The blockage of TSAP6 using ASO or siRNA-mediated knockdown induces cell death in *TP53* dependent manner [43]. An exosome discharge model came into existence due to the exosome secretory nature of TSAP6 [44]. The *TSAP6* KO was not efficient in making exosomes. TSAP6 transnationally controls the secretion of TCTP, and sometimes works as a detoxifier for the cells. Y2H assay revealed that TSAP binds with TCTP. Following TP53 activated, TSAP6 gets active and behaves as a tumor suppressor, and it was along with the *TPT1* gene found in the tumor revertants derived from U937 cell lines suggests that it is also an important gene involved in the process of tumor reversion [13].

#### KREV-1

The official gene symbol for *KREV-1* is *RAP1A* (Ras-related protein Rap-1A). *RAP1A* gene is located on 1p13.3. The *RAP1A* encodes the KREV-1 protein in humans*.* This protein is a GTPase. Overexpression of KREV-1 protein was found to be associated with different malignancies. Through AKT signaling, RAP1A promotes metastasis in esophageal squamous cell carcinoma (ESCC), [45] and an aggressive phenotype in colorectal cancer through PTEN/FOXO3/CCND1 pathway [46]. The revertants were obtained after prolonged exposure of prostate cancer cell lines with Azatyrosine. The revertants obtained were with elevated expression of KREV-1 showed low colony formation and no tumorigenicity in the mouse model; this suggests the role of *KREV-1* in tumor reversion [16].

#### MYC

MYC proto-oncogene, or bHLH transcription factor is encoded by the gene MYC (alias c-Myc) that is located on 8q24.21. *MYC* is a transcription factor that regulates a large number of genes essential for the progression of the G1 to S phase of the cell cycle. *MYC* is known to be associated with tumor progression in different cancers, including cervical, oral, and multiple myeloma [47–51]. MYC is very crucial for angiogenesis which is a key factor for the aggressive behavior of a tumor [51]. Tumor regression has been an important event observed in the large number of transgenic mouse models. The crucial question about the fate of cells remains unclear *i.e.* if they change to a non-malignant, malignant, or quiescent state. MYC inhibition leads to complete tumor elimination in lymphoma and osteosarcoma models [52, 53]. MYC inhibition reverses tumors into a normal or dormant state of cells. This puzzle was investigated in osteosarcoma and lymphoma where blockade of MYC successfully remove the complete tumor. Though intriguing, tumor cells derived from MYC-induced breast and hepatocellular carcinoma were reverted into a dormant state. Furthermore, dormant cells upon MYC reactivation again reverted to tumor state [54, 55]. This indicates the compulsiveness of MYC for the cells. MYC inactivation led to tumor reversion in different tumors [56]. As low as two-fold decrease in MYC expression can lead to tumor reversion in a cell-specific manner [57].

Tumor reversion was also observed upon MYC suppression in several malignancies, including T and B cell leukemia and lymphoma, squamous cell, and mesenchymal cancers [52, 53, 58, 59].

### Role of microenvironment and associated factors in tumor reversion

Interaction between tumor and microenvironment entities plays a crucial role in determining the behavior of the tumor. Apart from malignant cells, the TME cellular components are immune cells, the vasculature of the tumor, and the lymphatic endothelial cells, fibroblasts, adipocytes, and pericytes. The term “TME” sometimes is confusing giving the impression that only cancerous cells are involved. In contrast, non-cancerous cells are involved and those are essential for different stages including tumorigenesis, progression, and metastasis. Additionally, secretory proteins and blood vessels are also involved [60, 61]. Stromal cells and the extracellular matrix (ECM) constitute the structure of the TME. ECM consists of proteoglycans, fibrous proteins including collagen, fibronectin, laminin, tenascin, and hyaluronic acid [62]. TME has been reported in a large number of studies to play a very crucial role in tumor maintenance and progression. Furthermore, TME contributes to tumor reversion [63]. Tumor-associated macrophages (TAMs) are the cells found in TME and these are associated with the microvessel density of the tumor tissues. TAMs are mainly of two types based on their pro-tumorigenic (M2/Th2-activated) or anti-tumorigenic behavior (M1/Th1-activated). The balance between M1 and M2 decides the kind of phenotypic behavior expected in a tumor. The evolution of the TME depends on the stage and type of the cancer. TME can revert the anti-tumor program and favor a switch of infiltrated macrophages into an M2 phenotype with pro-tumor and immune-suppressive functions [64]. TAM-specific inactivation of IKKβ, which disrupts NF-κB signaling resulted in an M2-to-M1 switch, recruitment of natural killer cells, and subsequent tumor regression in an ovarian cancer model [65].

The M2 subtype of TAMs is crucial in creating an immunosuppressive TME because these macrophages can secrete cytokines, growth factors, and chemokines, that inhibit the immune checkpoints in the T-cells. This property has been exploited for reverting the tumor in several studies. TAMs are important in polarizing the phenotype of a tumor. The macrophage depolarization of an M2 phenotype via CSF-1R inhibition led to tumor regression of established high-grade gliomas [66].

The embryonic microenvironment plays an important role in reprogramming metastatic tumor cells [67]. The nodal inhibitor showed no effect in tumors treated alone than cultured within the vicinity of human embryonic stem cells (hESC). The latter not only responded but also the cells started initiated to show features alike normal phenotype [68].

The microenvironment plays an essential role in regulating tumor growth [2]. The cultured breast cancer cells treated with an antibody blocking integrin showed features like normal cells e.g. ductal structures looking alike normal breast epithelial cells [69].

It is also well established that the tumor progression in solid tumors rely on new vasculature formation through angiogenesis. Experimental evidences reveal that the most human tumors arise without angiogenic activity and remain dormant and viable as microscopic lesions for extended periods. The angiogenic phenotype in human tumors can also spontaneously revert to the non-angiogenic phenotype in the small population (~ 4–6%) of tumor cells. If the rate of reversion to the non-angiogenic phenotype can be increased therapeutically, this could lead to a novel anti-cancer strategy through tumor reversion.

### Alternative RNA splicing and tumor reversion

The process of RNA splicing is responsible for bringing diversity at transcript as well as protein levels. The RNA splicing machinery “spliceosome” orchestrates this process. RNA splicing has been reported to play a crucial role in different biological mechanisms essential for cancer progression, metastasis, tumor-microenvironment interaction, drug resistance, epithelial-mesenchymal transition (EMT), and mesenchymal-epithelial transition (MET), in tumor reversion. A peculiar characteristic of cancer cells is aberrant RNA splicing, which forces the cells to reorganize their specific RNA spliced forms required for that particular stress/cancer and contributing to the tumorigenesis. Cancer-specific AS events have been documented that are responsible for tumor progression as well [70]. The spliceosome complex controls the RNA splicing events. There are several cases where RNA splicing able to induce/modulate tumor reversion. Serine/arginine-rich splicing factor 1 (*SRSF1*) gene is also known as SF2/ASF. *SRSF1* upregulation has been reported in breast cancer. Notably, several endogenous splicing targets of SF2/ASF, including novel oncogenic isoform of the mTOR substrate, S6K1, are essential for SF2/ASF-mediated transformation. Also, RNA interference (RNAi) of SF2/ASF or the oncogenic S6K1 isoform resulted in the reversion of the transformed phenotype [71]. A number of macrolide splicing modulators (SPLMs) have been used for modulation of RNA splicing for anti-cancer activities. These SPLMs not only affect the total protein levels but also the PTMs (including but not limited to, phosphorylation, and glycosylation). Indole derivatives IDC92 have been tested for modulation of RNA splicing in breast cancer where these were able to show anti-proliferative activities in the cancer cells, and not only reverse the abnormal splicing form ΔRON (of proto-oncogene RON), but also the invasive phenotype of the breast cancer cells without altering the splicing of other targets like SF2/ASF [71].

In cancer cells, the pyruvate kinase muscle isoenzyme (PKM) plays a significant role in cancer cell metabolism to adapt to a new ambience. In a study on head & neck cancer cells, treatment with dietary-phytochemical able to induce reversion of PKM2 (cancer-specific isoform) into PKM1 (normal specific) isoform and also lead to inhibition of H&N cancer [72].

In the case of chronic lymphocytic leukemia (CLL), macrolide SPLMs like pladienolide-B and FD-895 were able to modulate the myeloid cell leukemia factor 1 (MCL1) gene transcript isoforms (larger-MCL1L: larger-anti-apoptotic, and shorter, MCLS: pro-apoptotic) after treatment in CLL-B cells. This impact was preferentially in CLL-B cells only [73]. Even derivatives of FD-895 were also able to recapitulate the same properties in primary CLL-B cells and other leukemia and lymphoma cell lines [74].

### Non-coding RNAs and their role in tumor reversion

In addition to high-throughput techniques like RNAseq have shown that the human transcriptome is complex and its regulation is controlled through different developmental stages [75]. The ncRNAs are functional as those are able to transcribe but unable to translate into protein. The ncRNAs primarily are of two types: small ncRNAs (< 200 nucleotide length) and long ncRNAs (lncRNAs, > 200 nucleotide length) [76]. Among sncRNAs are regulatory RNAs like microRNAs (miRNAs or miRs), rRNA, tRNA, & splicing RNAs [77, 78].

The miRs are single-stranded, endogenously occurring small RNAs with varying lengths from 20 to 23 nucleotides post-transcriptionally regulate gene expression [79]. An interaction via complementary base pairing between miR, and mRNA is essential for target mRNA’s translation or stability. These are involved in regulating the gene expression by integrating with the RNA-induced silencing complex (RISC). Further, miRs can suppress the translation or degradation by binding to the 3′-untranslated region (3′-UTR) of the mRNA [77]. In recent years, a different research group reported that the target of miRs is the 3′-UTR, and in some cases miRs target 5′-UTRs as well [80, 81].

### Role of miRs in tumor reversion

In most studies, miRs or microRNAs have been reported in tumor progression. Still, a few studies also reported the role of miRs in tumor suppression, and rare cases in reverting the tumor phenotype into normal (tumor reversion). Among miRs, miR-155 is a known macrophage polarization modulator. In the TAMs derived from the bone marrow-derived macrophages (BMDMs), overexpression of miR-155 was able to polarize tumorigenic M2 macrophages (anti-inflammatory profile) into inducing anti-tumor macrophage M1 (‘classic’ pro-inflammatory phenotype). The miR-155 expression was upregulated in M1 polarized macrophages types by > 120-fold suggesting its crucial role in reversing a tumorigenic to anti-tumorigenic phenotype [82]. Further, overexpression of miR-99b in TAMs educated them towards the anti-cancer phenotype that led to hindrance in the HCC and LLC growth and further improved immune surveillance [83].

The tumor could serve as a good target for inducing the tumor regression by targeting the genes using miRs or anti-miRs in cancers with altered protein-forming genes [84]. The miR-26a targeting c-MYC mRNA induced tumor regression in HCC [85]. Another miR let-7 was originally discovered first in nematodes has been reported in different malignancies. The let-7 group miRs are essential for apoptosis, cellular proliferation, and invasion of cancerous cells. The let-7 miR is essential in maintaining the state of differentiation in somatic cells. Ectopic overexpression of let-7 g in human ovarian cancer cell lines reduce cell’s growth, induces arrest of the G0/G1 phase of the cell cycle, reduces EMT and cell motility [86]. The reduced let-7 level was associated with regression in the mesenchymal phenotype and shorter survival. Higher let-7 expression and higher EMT could not form a detectable tumor, but in contrast, a lower let-7 level and lower EMT led to the tumorigenic phenotype [87].

The miRs have been associated with HCC, and a large number of differentially regulated miRs have been reported between HCC vs normal. Among these, miR-21 and miR-17-92 were subtly upregulated in HCC as compared with the normal tissue sample. The anti-sense oligonucleotide-mediated inhibition of miR-17-92 and miR-21 induced a significant reduction in cellular proliferation, which was ~ 55 and 21%, respectively. Additionally, considerable retardation was observed in the G1 phase of cell cycle in HepG2 cells. The knockdown of miR-17-92 also decreased the anchorage-independent growth significantly in HepG2 cells. Overall, this evidence shows that miR-17-92 knockdown led to partial phenotypic tumor reversion and suggests its involvement in tumor reversion [88].

The miR-200 family consists of very important miRs, including miR-200a, miR-200b, miR-200c, miR-141, and miR-429. The ZEB (zinc-finger enhancer-binding protein) / miR-200 response loop is a cellular plastic cell engine for the development and diagnosis. In particular, it can advance cancer toward metastasis by controlling the cellular stem cell culture. Interestingly, miR-200c induced overexpression overturns chemotherapy and EGFR-mediated resistance in reproductive cancer [89]. The miR-200c possesses pro-apoptotic properties and targets *FAP1* (an apoptosis inhibitor) and makes the cancer cells perceptive to apoptosis [90].

A systematic RNA screening led to the identification of miRs playing a key role backed by MET in the starting phase of phenotype reversion. These steps are directly dependent on miR-205 and the cluster of miR-200 family. The ectopic expression of the miR-200 family and miR-205 can revert the mesenchymal to epithelial transition (MET) in mesenchymal cells. In concordance with their EMT role, these miRs were lost in mesenchymal phenotype bearing invasive breast cancer cell lines [91].

Another important type of ncRNAs subtype is lncRNAs. We carried out an extensive search on it but unable to find even a single study reporting role of lncRNAs in tumor reversion. This is also largely possible because there is not even a single study focused on either deploying lncRNA or RNAseq profiling as both of these high-throughput techniques can capture the lncRNA profile between normal vs cancer groups.

### Transcription factors and tumor reversion

Alternative RNA splicing is the basis for bringing the diversity in the transcriptome and eventually in the proteome as well. An optimum balance between isoforms is critical for the normal biological functioning of different organisms. Besides AS, transcription factors (TFs) play a significant role in the regulation of different malignancies [92]. The TFs are involved in tumor regulation, proliferation, progression, and metastasis and play a significant role in regulating changing the phenotype from cancer to normal. Some of the TFs that have been reported in tumor phenotypic reversions are discussed here.

#### Homeobox D10 (HOXD10)

The homeobox D10 gene (*HOXD10*) belongs to the HOX family of genes that are important for various processes related to development [93]. HOXD10 has been reported to be involved in several different malignancies [94]. An elevated expression of HOXD10 led to tumor quiescence; it also has been reported as a TSG in pancreatic and cholangiocarcinoma [95]. HOXD10 induces reversion of tumor phenotype in 3D culture conditions in breast cancer [25].

#### Signal transducer and activator of transcription 3 (STAT3)

*STAT3* is an important transcription factor that consists of one SH2 domain and 3 CC motifs [32]. It is involved in various diseases, including cancers. It plays a pivotal role in inflammation, normal growth, and development. Under pathological conditions, its aberrant activation leads to growth, progression, angiogenesis, chemo-resistance, and tumor cell’s survival [96]. Blockage/inhibition of STAT3 showed a profound anti-cancer effect in vitro as well as in vivo conditions [97–99]. Targeting STAT3 by pharmacological or genetic means led to tumor reversion as well [100].

### Post-translational modifications in tumor reversion

PTMs are enzymatic modifications in proteins and play a very significant role in cell signaling. All the PTMs relevant to tumor reversion are summarized and discussed in Table 2. PTMs are the outcome of specific but local physiological or stressed/disease states. PTMs are critical events, which can alter the conformation of the protein, their stability, and diversity. PTMs are very crucial for biological processes, cellular proliferation, development, differentiation, diseases/tumor progression, and drug resistance [114]. PTMs are vital for dissecting the mechanistic angle of the biology behind tumor reversion, as we observed only a handful of PTMs concerning tumor reversion. Among PTMs, phosphorylation (p) had been a winner as it has been extensively studied for proteins such as EGFR in lung adenocarcinoma [115]. Studying phosphorylation (p) is tricky because serine (S), threonine (T), and tyrosine (Y) have varied ratios of 1000:100:1 [116]. Among these, studying pY is very difficult due to poor abundance in the system; therefore studying pY in any biological setting requires enrichment of the samples to capture the maximum possible events for pY specific PTMs. It can be achieved using pY specific anti-phosphotyrosine antibodies (Clone 4G10) [115]. In contrast, pS and pT residues can be enriched easily using Titanium Dioxide (TiO_2_) based enrichment method. Only one study was carried out in the multiple myeloma model of tumor reversion. The in vivo quantitative proteomics labeling technique called stable isotope labeling in animal cell culture (SILAC) was employed to identify the proteins of interest between revertant vs parental cells, and reported the significance of STAT3 in tumor reversion [21]. In SILAC, the cell line first cultured in heavy Arginine and Lysine that after sub-culturing for five passages leads to replacing of the unlabeled proteins by replacing light Arginine and Lysine. One of the significant differences between iTRAQ versus SILAC is that in the case of SILAC, there is a requirement of huge amount of protein. SILAC is performed on intact protein, and iTRAQ on the peptides.

**Table 2:** Post-translational modification, and other molecular events involved in tumor reversion

Name of Molecule	Gene Symbol	Post-translational / posttranscriptional Regulation	Type of Cancer	Drug Agents	Impact of PTM on Tumor Reversion	Reference
Retinoblastoma (RB)	RB1	Phosphorylation	Breast Cancer (MCF-7)	Axolotl Oocyte Extracts (AOE)	pRB and it’s nuclear localization reduced upon extract treatment, but not of CDKN1B (p27) in early hrs (6 h), but increased 12 h post-treatment. During reprogramming, induction of cell cycle arrest occurs during the process in oocyte extracts and it is stably maintained in treated tumor xenografts, and reprogrammed tumors showed reduced phosphorylation of RB at S780.	Saad et al 2018, [101]
CDKN1B	p27	Phosphorylation	MDA-MB-468 and MDA-MB-231 (Human breast cancer lines), and murine NIH/3T3 cells.	GGTI-2418	In normal cells, p27 inhibits nuclear Cdk activities and is thus considered a tumor suppressor. p27 expression is essential for tumor reversion. Phosphorylation at pY74 & pY88 of p27 activates p27 bound Cdk2/cyclin E complexes, which in turn transform p27 from an inhibitor of Cdk2 inhibitor to a Cdk2 substrate and eventually increases p27 expression.	Kazi et al 2009, [102]
GATA binding protein 1	GATA1	Phosphorylation and Acetylation	Cos 7 and NIH/3T3 Cells.	Tylase Inhibitor Trichostat in A (TSA).	Effect of different agents was tested on GATA-1 modifications in COS7 and NIH/3T3 cell line. Both SCF and erythropoietin elevated phosphorylation of GATA-1. The mutations in the phospho-site abrogated the phosphorylation shifts and protein turnover. Consistent with the idea that both acetylation and phosphorylation are required for turnover. The acetylation mutants were observed to be stable under mitogen stimulatory conditions and, moreover, that mitogen stimulation preferentially leads to the degradation of acetylated GATA-1.	Hernandez et al 2006, [103]
Protein kinase D1	PRKD1	DNA methylation	Breast Cancer cell lines (MDA-MB-231).	Bisulfite Sequencing	Inhibition of methylation of the PRKD1 promoter with DNA methyltransferase inhibitors can lead to re-expression of PKD1 and reversion of the invasive phenotype	Borges et al 2013, [104]
Glutathione S-transferase P1	GSTP1	Methylation	LNCaP human PCA cells	Procainamide	LNCaP PCA cells expressing GSTP1 appeared only after decreased GSTP1 promoter methylation (antagonistic to gene expression) after prolonged 5-aza-C exposure (for many generations), 5-aza-Ctreated LNCaP PCA cells that had unmethylated GSTP1 promoter expressing *GSTP1* mRNA as well as the corresponding polypeptides, regardless whether or not 5-aza-C present in the growth medium.	Lin et al 2001 [105]
RARB, CST6, CDKN2A and CCND2	RARB, CST6, CDKN 2A and CCND 2	Demethylation	MCF-7 & HCC1954 cell lines	Amphibian Oocyte Extracts ( AOE).	AOE induced higher demethylation levels for RARB and CST6 promoters than AOE, but CDKN2A and CCND2 showed similar levels of demethylation by either extract. Interestingly, embryonic stem cells extract (ESCE) can slightly induce RARB and CDKN2A demethylation, which is consistent with the inability of this extract to re-activate their expression. Demethylation of CGs from 8 to 13 was found for RARB, CGs 1–8, 14–18, 25 and 31 for CST6, CGs 1–7, and 21–28 for CCND2, and CGs 1–9 and 19–28 for CDKN2A.	Allegrucci et al 2011 [106]
Glycodelin (Progestagen Associated Endometrial Protein, PAEP or Placental Protein 14)	PP14	Deglycosylation	HEC-1B cells (ATCC HTB-113)	Glycodelin Isoform, Glycodelin-A (GdA)	Using enzymatic deglycosylation and/or differentially glycosylated glycodelin isoforms, it was observed that GdA help proliferating PBMC, trophoblast invasion was glycosylation dependent. Regardless of the differences in glycosylation between GdA and HEC-1B, both of them inhibited trophoblast invasion in a comparable potency.	Hautala et al 2020, [107]
Sialidase	NEU1		Murine melanoma variant B16-BL6 (B16 murine melanoma)	Lysosomal Sialidase	Sialidase impact was studied by using *i.v.* in syngeneic C57BL/6 mice, as well as in cell lines. In cell line, a reduction in metastasis from 40 to 76% was observed in the sialidase transfectants. The metastatic nodules in the transfectants reduced in comparison to the control. Reduced lung metastasis was seen in athymic BALB/c nude, and SCID mice suggested the role of immune system in weakling of the metastasis.	Kato et al 2001, [108]
Focal adhesion kinase 1	FAK	Phosphorylation	Lewis Lung Carcinoma cells (LLC)	FAKY861	Vessel regression can be determined and the study used the mouse model and concluded that the pY861 site phosphorylation is crucial in blood vessel regression in the tumor phenotype in Pdgfr b Cre+; FAKY861F/Y861F mice.	Lees et al 2020 [109]
Tumor Protein TP53	P53	DNA methylation	K7M2 and K12 (murine osteosarcoma) cell populations with differing metastatic potentials (K7M2 is highly metastatic to the lung but K12 is less metastatic)	Chick Embryo Extract (CEE)	CEE extract able to revert the DNA methylation *p16*, *TP53*, and *ECAD* genes. Further, these results were corroborated by the PCR assay as mRNA upregulation was observed post-CEE treatment. The demethylation induced by CEE promotes expression of the above-mentioned genes, and led to reversion of the metastatic phenotypes in highly metastatic osteosarcoma cells.	Mu et al 2014 [110]
Ganglioside Monosialic 2 / Ganglioside Monosialic 3	GM2/G M3	Silylation / Phosphorylation	Bladder cancer cells	Anti-GD2 monoclonal Antibodies	GM3 or GM2 gangliosides induced reversion from oncogenic to normal phenotypes via formation of a complex of tetraspanin CD9 or CD82 in the microdomains. Once GM3/CD9/integrin α3 complex formed in Bladder cells, tumor cells’s phenotype suppressed. GM2 and CD82 together inhibits tyrosine kinase activity upon interaction with MET. Also the cross talk between integrin with Met33. Gangliosides with high levels of silylated. Gangliosides control integrin α5β1-mediated epithelial cells adhesion to FN (fibronectin) via carbohydrate-carbohydrate interactions.	Tsuchida et al 2018 [111], Furukawa et al 2012 [112]
Cyclin-Dependent Kinase Inhibitor 2A (CDKN2A)	CDKN2A or P16	Demethylation	Different tumor types including gastric, colonic, ovarian, breast, renal, lung cancer & melanoma.	DNMT1 Inhibitor (MG98)	MG98 is an anti-sense oligonucleotide binds to 3′-UTR of DNMT1 in human cells, and that results in demethylation of selected genes by reducing the CpG island and allowing the tumor suppressor genes to re-express.	Plummer et al 2009 [113]

Phosphorylation of RB1 inhibited at S780 residue in MCF-7 cell line after treatment with axolotl oocyte extract, and subjected to cellular reprogramming and arrest of the cell cycle as well. Furthermore, inhibition of CDK activity, and reprogramming of tumor cells occurred which leads to cell cycle arrest. The in vivo reprogrammed tumors in mouse xenograft showed a decrease in the pS780 levels of RB1 protein [101].

In breast cancer cells, the p27 expression is essential for tumor regression. Upon treatment with GGTI-2417 (a methyl ester prodrug that is a selective inhibitor of GGTase I), an accumulation of p27 protein occurs in G0/G1 phase, followed by induction of apoptosis in breast cancer cells. An accumulation of nuclear p27 in an in vivo mouse model led to regression of the tumor compared with the controls [102].

Glycosylation is another significant PTM event that has been observed in ~ 50% of the proteins. It has been reported about tumor progression in the large number of studies. There are selected studies that are only available on the role of glycosylation in association with tumor reversion [107]. Glycosylation is mainly of three types based on glycosidic linkage: O-, C-, and N-glycosylation.

### Role of animal models in tumor biology

The animal models are integral part of the cancer therapeutics-based studies. The tumor models for different cancers have proven to be very helpful not only in understanding the disease biology but also for the development of novel therapeutic targets. The characteristics of these mice combine the features of the NOD/ShiLtJ background, the severe combined immune deficiency mutation (*SCID*), and IL2 receptor gamma chain deficiency that results in lack of mature T, B, or functional NK cells, and are deficient in cytokine signaling in NSG mice leading to better engraftment of cells of interest. Some of the important animal models including SCID or transgenic used for different cancers other than tumor reversions are summarized in Table 3.

**Table 3:** Animal models used for different malignancies in studying tumor biology (other than tumor reversion)

Model Type	Malignancy	Phenotype	References
CC10-rtTA; (tetO7)CMV-K Ras^G12D^ (Transgenic)	Lung Cancer	Bronchogenic adenocarcinomas. Phenotype is completely reversible upon Dox removal.	Fisher et al 2001 [117]
KPC Mouse model	Pancreatic Adenocarcinoma	It develops important key features observed in human PDA including pancreatic intraepithelial neoplasia alongside a robust inflammatory reaction including exclusion of effector T cells. KPC mouse contains a conditional point mutation in the transformation related protein 53 gene TP53R172H), and a point mutation in KRAS gene (KRASG12D) both of which generate non-functional proteins.	Hingorani et al 2005 [118]
NSG mice (NOD.Cg-*Prkdcscid Il2rgtm1Wjl*/SzJ) used for making human esophageal tumor xenograft using TE11 cell line	Esophageal squamous cell carcinoma	Subcutaneous treatment with pharmacological inhibitor entospletinib (GS-9973) for 10 days led to reduction in tumor growth by 55%.	Barbhuiya et al 2018 [119]
NOD-SCID mice implantation with MDA-MD-231	Breast Cancer	hMAb173 treatment led to 60% reduction in the TNBC tumor growth compared to the control group. The microscopic study revealed that hMAb173 treatment effectively degraded AXL in tumor cells.	Wu et al 2015 [120]
Eμ-Tcl-1 transgenic mouse model	Chronic lymphocytic leukemia	The TCL1 gene of human origin under the control of the immunoglobulin heavy chain variable region promoter and immunoglobulin heavy chain enhancer (Eμ-Tcl-1). The model is time consuming due to disease delayed development, and TCL1 overexpression does not allow relextion of the genetic complexity of CLL.	Bichi et al 2002 [121]
human/mouse radiation chimera	CLL	Transplantation of CLL PBMC into peritoneal cavity of irradiated Balb/c or BNX mice, radio-protected with bone marrow from SCID mice.	Shimoni et al 1997 [122]
NOD/SCID	CLL	Transplantation of CLL PBMC in NOD/SCID mice and combining intravenously and in transperitoneally injection.. However, these mice still retain normal natural killer (NK), and myeloid cells, and these cells were likely responsible for interfering with the in vivo engraftment of some human leukemia’s/lymphomas.	Durig et al 2007 [123].
Transgenic mice model with human MET in hepatocytes under the control of tetracycline	Hepatocellular carcinoma	In this study, early deaths prevented by feeding the mating parents and newborn pups doxycycline to repress expression of the MET transgene. Continued expression of MET is required for maintaining HCC.	Wang et al 2001 [124]
Transgenic mice model expressing *KRas4b*^*G12D*^ under the control of doxycycline (a form of tetracycline)	Lung adenocarcinoma	DOX induction after two months led to development of adenoma, and adenocarcinoma of lungs, but removal of DOX in contrast caused rapid downregulation of mutant KRas RNA and auxillary apoptotic regression of an early proliferative lesions as well as tumors.	Fisher et al 2001 [117]
K14-rtTA/TetRE-ErbB2 ‘Tet-On’ bitransgenic mouse system	Skin carcinoma	Until ErbB2 expression induced by doxycycline (Dox), the animals were normal, but prenatal induction led to death. Skin hyperplasia observed in animals after two days, and Dox withdrawal reverted these changes to normal.	Xie et al 1999 [125]

### Role of chemical biology, and anti-therapeutic agents in tumor reversion

**Fig. 4:**
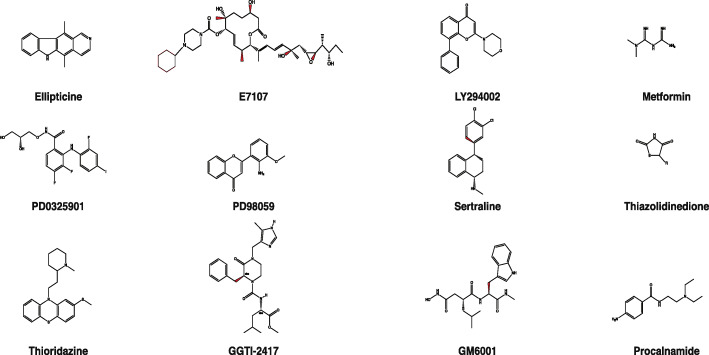
The chemical structures of compounds used for induce tumor reversion. A number of compounds have been used for reverting the phenotype of a tumor into normal. The structure of the following compounds have been drawn here using ChemDraw: Ellipticine, E7107, LY294002, Metformin, PD0325901, PD98059, Sertraline, Thiazolidinedione, Thioridazine, GGTI-2417, and GM6001

In addition to the availability of several cell lines and animal-based models, chemical probes have been reported to induce tumor reversion. These include not only antibodies but also natural product-based compounds such as ellipticine, thioridazine, E7107, sertraline, metformin, and thiazolidinedione (Fig. 4). Selected compounds have been discussed below, as these have been directly or indirectly impacted the process of tumor reversion.

#### Ellipticine

Ellipticine is a natural product of pyridoindole alkaloid naturally derived from the leaves of *Ochrosia elliptica* and *Rauvolfia sandwicensis*. Ellipticine’s anti-cancer activity has been reported in a number of malignancies [126]. It can induce phenotypic reversion in tumor cells at non-cytotoxic concentrations in the cell lines [127].

#### Thioridazine

It is an antipsychotic drug that consists of antimicrobial activity. It can restore doxorubicin sensitivity in leukemia as well as in multidrug-resistant T-lymphoma cell lines and capable of inducing apoptosis in these cells [128]. It has been used as a tumor reverting agent because it inhibits TCTP [129].

#### Metformin

Metformin is prescribed as the first line of treatment for type 2 diabetes [130]. It is capable of stimulating cell survival and mitogenesis in many cancers including breast, liver, colon, pancreas, and skin [131]. It has been shown to reduce cancer by ~ 57% in T2DM patients [132]. Metformin stimulates adenosine monophosphate (AMP)-activated protein kinase. AMPK can be directly activated by an increase in the ratio of AMP: ATP in metabolic stress including hypoxia and glucose deprivation [133]. MCF10ADCIS cell line is ductal carcinoma in situ models of breast cancer. It is capable of making irregular, large spheroids without a lumen. Still, treatment with metformin induces luminal-like morphology and also reverses overexpression of markers such as VIM, FN1, and CDH2 suggesting the role of metformin in tumor reversion.

#### Thiazolidinedione (TZD)

It has been used for the treatment of type 2 diabetes. TZD’s phenotype treated anaplastic thyroid carcinoma cells changed to epithelial-like cell morphology. It is a typical feature observed in the differentiation of epithelial cells of thyroid origin, and also in the reversal of EMT [134].

#### Sertraline

It is an antidepressant drug that is an inhibitor of CYP2D6 and CYP2B6 in vitro [135]. Sertraline is used to treat non-small cell lung cancer (NSCLC); it inhibits the viability of NSCLC cells and shows synergy with erlotinib. Sertraline also has been used in the treatment of breast cancer [136]. Sertraline is used with thioridazine in cancer treatment and tumor reversion by targeting the major driver protein TCTP [129].

#### LY294002

LY294002, a morpholine-based compound that is a powerful inhibitor of phosphoinositide 3-kinases (PI3Ks) [137]. LY294002 in coordination with DAPT (γ-Secretase Inhibitor) inhibits Notch1, HES1, and pAkt in gastric cancer cells, thus inhibit metastasis of gastric cancer through mutual enhancement [138]. LY294002 and baicalein inhibit cellular proliferation and induce apoptosis in liver cells via the PI3K/Akt signaling pathway in combination with baicalein [139]. LY294002 combined with PI3K inhibitor and dibutyryl-cAMP led to tumor reversion in mammary tumor cells through cellular reprogramming of cell polarization and morphogenesis from tumorigenic to normal [140].

#### PD0325901

It is also called Mirdametinib, non-ATP-competitive MEK I with IC_50_ of 0.33 nM in cell-free assays, roughly 500-fold more potent than CI-1040 on phosphorylation of ERK1 and ERK2. PD0325901 showed its potential in converting the tumor phenotype into the normal in an isogenic cell line model derived from MCF10A [28].

#### E7107

E7107 is a macrolide that is a 7-urethane derivative of pladienolide D (PLAD-D). Like pladienolide-B (PLAD-B), spliceostatin, Herboxidiene, and Trichostatin A; E7107 targets SF3B1 protein that is part of U2 snRNP of the spliceosome complex. The spliceosome modulator, E7107 reverses cancer aggressiveness and inhibits castration-resistant prostate carcinoma in xenograft and autochthonous prostate cancer models. Treatment of LNCap (prostate cancer cell line) with E7107 led to changes in the transcriptome, which are more like normal cells, indicates that E7107 modulates the transcriptome via modulation of spliceosome machinery by binding to the SF3B1 protein. This suggests that RNA splicing machinery also plays a vital role in the process of tumor reversion [141].

### In vitro, 3D-culture-based, and in vivo models for studying the tumor reversion

The 3D models mimic more closely to in vivo behavior of cells; therefore, many studies using breast cancer as a preferred model were carried out using the 3D technique. Cancer cell culture in 3D, material, or embryonic fields fortifies the TOFT anecdotal through the microenvironment’s ability to overcome the mutated genes’ activity and promote the malignant phenotype’s reversion. The co-culture of cancer cells with normal cells of the microenvironment can guide cells into a normal phenotype through a process of reversion via the restoration of a normal, and strong morphogenetic field [12, 142]. Additionally, 3D culture models have been used where reconstructed, but normal tissue architecture mimicking biological microenvironments was used and the tumor cells successfully novitiate the normal tissue architecture [143]. Further, these changes make these reversed cells prone to apoptosis and differentiation, and at last culminate the reprogramming of “normal” phenotype [2, 67]. Tumor reversion has been studied in vitro, in vivo, and 3D-culture-based models. A summary of all these models is presented in Table 4.

**Table 4:** In vitro, 3D culture, and in vivo models used for studying tumor reversion

Model Systems	In vivo/ 3-D / In vitro	Cell Line/Tissue	Observation regarding tumor reversion	Drug Agent/Viral strain	Reference
MSV transformed 3 T3 cell line based Mouse Model	In vivo	Murine Sarcoma Virus transformed mouse 3 T3 cells	Murine sarcoma virus-transformed mouse NIH/3T3 cells (negative for the murine leukemia virus) give rise to a sarcoma virus upon superinfection of murine leukemia virus. The revertants support leukemia virus growth and show enhanced sensitivity to murine sarcoma superinfection and, like normal cells, do not release RNA-dependent DNA polymerase activity.	Murine Leukemia Virus (MuLV)	Fischinger et al 1972, [144]
Twenty four acute promyelocytic leukemia (APL) patients	In vivo	Human APL	APL patients treated with all-trans retinoic acid attained complete remission without developing bone marrow hypoplasia, and a gradual terminal differentiation in bone marrow derived tumor cells was observed as evident due to presence of Auer rods in mature granulocytes, followed by the re-emergence of normal hematopoietic cells upon remission.	Retinoic Acid (45 to 100 mg/m^2^/day)	Huang et al 1988, [145]; Zhou et al 2007, [146]
Transgenic Mice Model (MTA transgene)	In vivo	Mice carrying the tetracyclineresponsive trans activator (tTA) gene (MTA transgene)	Controlled expression of simian virus 40 T antigen (Tag) in the submandibular gland of transgenic mice four months from the time of birth induced cellular transformation and extensive ductal hyperplasia. Silencing of Tag led to reversal of hyperplasia for 3 weeks, but NOT after seven months as hyperplasia persistence was observed. Reversal of ploidy in ductal cells was possible in 4 months old animals, and observed to remains polyploidy at the age of 7 months even when devoid of TAg.	Simian Virus 40 (SV40)	Ewald et al 1996, [147]
BCR–ABL1-(tTA) induced Double Transgenic Mice Model	In vivo	Double transgenic mice (BCR–ABL1-tetracycline transactivator (tTA))	Withdrawal of tetracycline administration from double transgenic animals (BCR–ABL1-tetracycline transactivator (tTA) permits BCR-ABL1 expression and cause lethal leukemia. The rapid disappearance of leukemic cells from the blood suggested apoptosis rather than differentiation as the underlying mechanism for reversing the phenotype in this model. A constitutive BCR–ABL1 expression is crucial for maintaining the cancer of hematopoietic system and phenotype of the leukemic cells is completely reversible at the advanced stages of the disease.	Withdrawal of Tetracycline	Huettner et al 2000, [148]
Polyoma transformed hamster embryo cell model	In vivo	Hamster embryo cells (transformed by polyoma virus inoculated with large plaque virus LPll)	Hamster embryonic cell transformed using polyomavirus after inoculating with large virus LP-II plaque. A high frequency of variants with reversion phenotype observed in transformed cells.	Carmine	Rabinowitz et al 1968, [149]
MGI (macrophages and granulocyte inducer) grafted mice model	In vivo	Human and mice myleoid leukemia cells	Chromosomal studies of MGI + D+ leukemic cells showed that all the chromosomes could still be persuaded to develop a normal differentiation phenotype without a completely normal complement. Reversion of the phenotype of malignant cells with standard growth control without a completely normal chromosome complement was observed in other cell types as well.	Grafting of MGI producing cells	Sachs et al 1978, [150]
SCID Mice for making tumors derived from K562 and KS cells	In vivo	K562 leukemia cells and the clone KS resistant to cytopathic effect of H1 parvovirus	H-1 parvovirus preferentially kills neoplastic cells, and therefore used for selecting cells with the suppressed phenotype. KS (a cell line derived from K562) resistan to cytopathic effect of the H-1 virus displays a suppressed malignant phenotype. This and cellular resistance to H-1 killing appear to depend on the activity of wild-type p53.	H-1 parvovirus	Telerman et al 1993 [151]
3D In vitro Culture system ‘on top’ and disease-on-achip (DOC)	In vitro, DOC model to mimic TME.	Pre-invasive (S2 cells) and invasive (T4–2 and MDA-MB-231 Cell line)	The DOC model used for the breast cancer cells with varied tumorigenecity. In the 3D culture DOC model to mimic TME, the complex treatment had non-toxic effects on S2 cells, but induce significant cytotoxicity in invasive cells. Also, in 3D model, the cells were able to produce breast ductal architecture. Treatment also abolished tumor phenotype of invasive cells downregulation of markers such as EGFR, P50, NFκB and β1-integrin was observed.	e trans-[Ru(PPh3) 2(N,Ndimethyl-Nthiophenylt hioureatok2O, S)(bipy)]PF6 complex	Becceneri et al 2020 [152]
3D culture model to test RADARADARADARADACONH2 (RADA16)	In vitro, Effect of RADA on CD44+/ CD24-cells derived from breast cancer cell line	CD44+/CD24-sub population selected after sub-culturing MDA-MB-435S	MDA-MB-435S was enriched in CD44+/CD24-phenotype expressing cells. As compared with matrigel and collagen I, cells cultured in 3D RADA16 nano-fiber scaffold showed reversion of tumor phenotype, and formed round colonies and well-organized centric nucleus with regular morphology of the cells.	Cancer cells tested by culturing in 3D model either in RADA16 nanofiber scaffold versus Matrigel collagen I	Mi et al 2015 [153]
3D model using Engelbreth-Holm-Swarm extracellular matrix extract (EHS) matrigel and rat-tail collagen	In vitro, Cells embedded in the EHS Matrix as single cells allowed to grow for 10–121 days	Human breast epithelial cell line (S1), and invasive (T4–2 cell line)	Blocking of the β1-integrin using anti- β1-integrin MAB (clone AIIB2), T4–2 cells resistant to the antibody, but developed morphology very much alike S1 cells.	anti- β1-integrin MAB	Weaver et al 1997 [154]
3D laminin-rich basement membrane (3DlrBM) model for studying human breast cancer	In vitro	Breast (MDA-MD-231), and cervical cancer cell line (HeLa)	The retroviral expression transferred to re-establish HoxD10 expression in the malignant breast tumor cells. A phenotypic reversion after decrease in the expression levels of α3 integrin was observed along with decelerated cellular proliferation.	Retroviral gene transfer to restore HoxD10 expression	Carrio et al 2005 [25]
3D culture system for studying human breast cancer	In vitro	Epithelial cell line (HMT-3522), and breast cancer cell line (T4–2)	Downregulation of EGFR and β1-integrin observed in breast tumors and normal cell line upon treatment with an antibody against β1-integrin function-blocking mAb. This further led to growth arrest and tumor phenotype changes, looking more like normal breast cell morphology.	β1-integrin function blocking mAb	Wang et al 1998 [155]
Biodegradable meshwork (Hyalograft 3D): primary breast cancer cells cultured in 3D collagen-I gels both as mono- and as co-culture with human mammary fibroblasts (HMFs).	In vitro	PBCs and HMFs were used in the ratio of 1:2. Primary breast carcinoma cells	In a total of 38% of the cases, reverted tumor phenotype was observed. Presence of acini formation was observed as a conversion characteristic into the normal phenotype, which was reported to be 2–7 fold, and glandular structures were observed in reverted co-cultured cells. In the isolated primary breast carcinoma cells; out-of 13, only 5 exhibited reversion of their malignant phenotype. Also, differentially expressed genes were identified such as ELF5, MAL, SQLE, MAP6, and ZMYND11.	PBC cultured alone or with HMFs for comparative analysis	Romer et al 2013 [156]
3D basement membrane culture model	In vitro	Mammary epithelial cells (MCF-10A cell lines)	RNAi mediated inhibition of Bim expression blocks the luminal apoptosis and slow down the formation of lumen.	RNAi to block Bim expression	Reginato et al 2005 [157]
Human breast 3D tissue morphogenesis models	In vitro	Human breast cancer MCF7 cells	MCF7 cells were co-cultured with primary human breast fibroblasts. The presence of normal breast fibroblasts constitutes the minimal permissive microenvironment to induce near-complete tumor phenotypic reversion.	Cells alone or cocultured with primary human breast fibroblast cells	Krause et al 2010 [158]
3DlrECM	In vitro	Breast cancer cell line T4–2	Small molecule inhibitor of TACE, TAPI-2, reverted the malignant phenotype of T4–2 cells into phenotypically normal mammary acinus like architectures. The TACE-dependent shedding of amphiregulin and TGF-α was also observed in several additional breast cancer cell lines.	Small molecule inhibitor of TACE, TAPI-2	Kenny et al 2007 [159]
Kirsten sarcoma virustransformed NIH/3T3 cell line model	In vitro	NIH/3T3, DT (Ki/HGPRT- NIH/3T3), Ki/TK- NIH	Flat revertants with in vivo reduced tumorigenicity isolated from populations of 3 T3 cells transfected with a cDNA expression library derived from normal human fibroblasts.	Kirsten sarcoma virus	Noda et al 1989, [160]
Lysosomal-type sialidase b16 melanoma cells Murine model	In vitro	B16-BL6 murine melanoma cells	Lysosomal sialidase overexpression inhibits the metastatic potential of B16 melanoma, at least partially through reduction of cell growth and sensitization to apoptosis. This shows sialidase is involved in cellular functions, which are affecting malignancy characteristics of cancer cells.	Lysosomal-type sialidase	Kato et al 2001, [108]
GM3-mediated cell line model	In vitro	KK47 (noninvasive & nonmetastatic) and YTS1 (highly invasive and metastatic)	Ganglioside GM3 expression was higher in KK47 than in YTS1 cells. GM3 shows multiple functions like Integrin α3 with CD9 have more vital interaction. RNAi was used to knockdown CD9 that gives high cell motility. An addition of GM3 exogenously induces reversion of high motility YTS1 to low motility phenotype. An increased level of GM3 suppresses the motility as well as invasiveness of the tumor cells.	Gangliosid es GM3	Mitsuzuka et al 2005, [161]
Viral oncoprotein Jun (v-Jun) Fibroblast cell line model	In vitro	Mouse fibroblast cell line C3H 10 T1/2 & the chicken fibroblast cell line DF1	Upregulation of GM3 synthase occurs upon GM3 transfection in v-Jun-transformed 10 T1/2 cells, and this reverts the oncogenic phenotype into normal as indicated by anchorage-independent growth.	Sialosyllac tosylcera mide	Miura et al 2004, [162]
Human breast epithelial cells	In vitro	MCF10A Treated with or without PD032590 (MEK inhibitor)	MCF10A treatment with MEKi showed phenotypic reversion as well as downregulation of different molecules involved in membrane transport, metabolism, cell adhesion, & downregulation of biological processes crucial for tumor-related phenotypes including cellular proliferation & metastasis	MEK Inhibitior	Leung et al 2020, [28]

### OMICS approaches for solving the puzzle of tumor reversion

Multi-omics approaches contributed to understanding the multi-factorial diseases like cancer, diabetes, stroke, essential hypertension, and meningitis [19]. This became possible due to some of the landmark discoveries that lead to the development of techniques such as transcription activator-like effector nucleases (TALEN), zinc finger nucleases (ZFNs), and clustered regularly interspaced short palindromic repeat (CRISPR) [163].

### Nuclease based genome editing techniques, and shRNA screening

Unlike CRISPR, which can introduce multiple gene mutations concurrently with a single injection, TALENs are limited to simple mutations. CRISPR transfections also have higher efficiency, whereas TALEN editing often results in mosaicism, where a mutant allele is present only in some of their transfected cells [164]. For selecting TALEN nuclease sites, T must be before the 5′- end of the target DNA sequence. The shRNA vectors generally provide high cell-to-cell uniformity within the pool of treated cells and very consistent results between experiments. In contrast, CRISPR and TALEN produce results that are highly non-uniform from cell to cell due to the stochastic nature of the mutations introduced. Not in viruses and eukaryotes, but in prokaryotes, the CRISPR/Cas system can be found naturally. The enzymatic activity of the Cas9 protein is comparable to anchor scissors. Using CRISP-Cas9, gene editing can occur anywhere in the genome, as long as the short guide RNA (sgRNA) binds upstream of a PAM sequence. The sgRNA is a fusion of crRNA and tracrRNA [165].

#### ZFNs

ZFNs were the first custom DNA endonucleases, which could recognize 3–4 bps sequences and cleave the target DNA. Each zinc finger is typically made up of approximately 30 amino acid modules and is capable of recognizing 3 to 6 nucleotide bases. Since ZFN is a heterodimer (it is composed of a zinc finger domain and a Fok1 endonuclease domain), the endonuclease domain must dimerize before it can create a double-strand break in the DNA. However, this automatically happens as it recognizes the binding site. The Fok1 nucleases are also activated at this point. After cleavage, the cell then tries to repair the breakage, either through non-homologous end joining (NHEJ) wherein it seals the two ends of the DNA back together, or via homology-directed repair wherein it uses a copy of the gene sequence to fix the break, thereby incorporating the desired sequence into the DNA [166]. Using ZFNs, major challenge is to predict the specificity of the final arrangement, as Zinc fingers are known to influence neighboring fingers’ specificity.

#### TALENs

TALENs are fusion proteins (composed of a bacterial TALE protein and Fok1 endonuclease) whose specificity is derived from the protein-DNA association. It is normally comprised of 33–35 amino acid modules, each targeting a single nucleotide. Thus, by assembling different TALEN moieties, researchers can recognize any specific DNA sequence they like. Compared to ZFNs, TALENs are cheaper and produce faster results. They are also more flexible and easier to design due to their well-defined target specificities (the activity of each TALE does not affect the binding specificity of neighboring TALEs). However, both techniques are not limited to mutagenesis in mouse embryonic stem cells and have been successfully used to engineer modifications in several animal and insect species (e.g. zebrafish, rats, livestock, fruit flies, monarch butterflies, and nematodes). The note of caution is that TALEN motifs are also linked with Fok1 endonuclease, so dimerization is required before it can successfully cleave the DNA [167].

#### Clustered regularly interspaced short palindromic repeat/CRISPR associated 9 (CRISPR/Cas9)

CRISPR/Cas9 is an RNA-based bacterial defense mechanism composed of two types of RNA (one being the trans-activating crRNA and a single guide RNA) and Cas9 endonuclease. While the other two systems are both man-made, the CRISPR/Cas system is derived from bacteria. In nature, the CRISPR system is activated when a virus or foreign pathogen invades a bacterium. With the help of the appropriate Cas proteins, the system captures and cuts a portion of the viral DNA and incorporates it into the CRISPR locus of the bacterial genome [168]. When the same virus attacks the bacterium, the CRISPR loci produce guide RNA (gRNA), which takes the Cas proteins to the matching target sequence in the viral DNA. The Cas proteins then bind to the target and cleave the viral DNA at a specific location, rendering it inactive. Compared to ZFNs and TALENs, the CRISPR/Cas9 system is undeniably simpler, cheaper, and more efficient [165]. Notably, the CRISPR/Cas9 system can be designed for any genomic targets and multiplexed by adding multiple gRNAs.

Genome editing advanced techniques such as CRISPR could be exploited in addition to the classical human H1 parvovirus mediated generation of revertants. CRISPR was successfully used for genome editing of the MDA-MB-231 cell line to convert its aggressive phenotype into a mild one [29]. CRISPR could be used for generating models for cancers that have not been studied so far by focusing on tumor reversion. CRISPR allows creating a mutant or knockout of a particular gene without much hassle.

### Target discovery using shRNA screening

The major difference between shRNAs and siRNA is that shRNAs can stably integrate into the genome through virus-mediated transduction, but siRNAs transiently expressed in the cells. siRNA sequences between 19 and 29 nt are generally the most effective. Between siRNAs and miRs, siRNAs originate predominantly from exogenous dsRNA, but in contrast, miRNAs originate from the genome of the cell. siRNA-mediated gene silencing represents a cell defense mechanism against exogenous dsRNA, and miRNA-mediated gene silencing is an integral gene expression regulation process. In the case of siRNA, it is generated when the double-stranded RNA cleaved by a nuclease called Dicer. Inactivation of RNA using siRNAs referred to as RNA interference. TCTP has been studied using shRNA in breast cancer where inhibition of TCTP by shRNA led to induction in the expression of TP53 with a significant decrease in sphere-forming efficiency [169]. Another good example where lipogenesis was suppressed using shRNA by targeting FASN in breast cancer which led to tumor reversion [30]. Screening the shRNA library could help us in identifying the targets for tumor reversion as it was done in some other conditions such as reversion of multidrug resistance in some cancers.

The gene expression plays a vital role in transforming the phenotype of the malignant cells into normal cells. Techniques like RNAseq could be crucial in delineating the alternative RNA splicing events to identify differentially regulated AS events such as intron retention, exon skipping, alternative 3′SS, and 5′SS between normal vs. cancer cell types. Furthermore, RNAseq can help to identify crucial lncRNAs playing a significant role in tumor reversion, as we were unable to find even a single study-reporting role of lncRNAs involved in tumor reversion.

Among multi-omics techniques, proteomics-based techniques are crucial as they provide the landscape of overall proteome and shed light on changes in the PTMs [101, 102, 115].

Suppose we want to study and understand tumor reversion in a better way. In that case, we must explore different cancer-specific models using the high-throughput multi-omics study to make a compendium of differential proteins regulated involved in control as well as in the regulation of the process of tumor reversion.

The cell line models with various tumorigenicity levels could be ideal for dissecting the mechanism of tumor reversion. Among those, the MCF10A cell line with other variants like pre-neoplastic (MCF10A-T1K or T1K), MCF10CA1h or CA1h (low-grade), and MCF10CA1a or CA1a (high-grade) could be used for exploration of tumor reversion by implementing in vitro proteomics techniques, [170] such as isobaric tagging for absolute quantitation (iTRAQ) coupled with LC-MS/MS. Interestingly, this is the only study so far carried out in any of the malignancies to study tumor reversion. These kinds of models have the advantage of prohibiting genetic variants’ entry because all these are in the same genetic background. Furthermore, the 8-plex iTRAQ quantitative proteomics labeling technique allows running the technical replicates in parallel i.e., a maximum of eight samples can be compared and analyzed [171]. The iTRAQ technique has advantages in terms of the usage of less amount of protein (50 μg) compared with other methods. It enables quantitation of proteins and peptides by labeling the samples with isotope encoded reporter ions.

### Small molecule library screening in tumor reversion

Small molecule library screening in a high-throughput manner could help in the discovery of chemical leads that are the potential starting point in the tumor reversion process. HTS led to the identification of a novel p110-δ inhibitor that accelerates the anti-myeloma effect of bortezomib [172]. This facilitates rapid evaluation of thousands of small molecules in physiologically and biologically relevant assays for tumor reversion. The H1 parvovirus-based cell line models of tumor reversion could be ideal for HTS to identify the lead small molecule with the potential to induce tumor reversion.

### Challenges and future perspectives

Amongst reverting the phenotype approaches, reversion of M2-like TAMs to the tumor-suppressive phenotype by modulating the TME is the most promising one because phenotypes of macrophages are highly sensitive to TME stimuli [173].

In vitro and in vivo labeling techniques coupled with LC-MS/MS must be applied to find differentially regulated proteins crucial for cellular reprogramming and tumor phenotype reversion. Additionally, these high-throughput techniques could help in identifying the PTMs crucial for this biological process. Also, studies have shown that proteins with basic nature such as histones are prone to PTMs, and various studies have shown that miRs can target and regulate histones at specific positions [174].

A number of studies directly or indirectly supporting the fact that it is possible to change (complete or partially) the morphological behavior of the tumor cells exactly or nearly like a normal cell. Some of the difficult questions still need to be addressed if tumor reversion can be recapitulated among all types of malignancies. Will it be an alternative to treatment options like chemotherapy in the near future? The tumor regression model ideally in the same genetic background could be the best starting point using quantitative proteomics approaches such as SILAC and transcriptomics approach using RNAseq to address the significantly altered changes at protein and RNA levels. It is important from a therapeutic perspective that it works at the molecular level and drives a cancer cell to lose its malignancy by halting the tumor progression. So activating the tumor reversion pathway or mimicking, could be a promising potential treatment option for cancer.

## Conclusion

The molecular biology behind the process of tumor reversion is not only interesting but intriguing as well. The experimental evidence from different studies clearly suggests the usefulness of handful of molecules, including miR, post-transcriptional events in certain genes, and proteins with associated PTMs as potential agents for phenotypic tumor reversion. To date, making tumor reversion, as a treatment option remains a dream, but evidences, all together suggest more molecular layers derived from in-depth analysis using multi-omics approaches and shreds of evidence, all together suggests that this dream could become a reality in the near future.

## Supplementary Information

**Additional file 1.**

## Data Availability

All data generated and analyzed during our study are included either in the published article or supplementary data associated with the manuscript.

## Abbreviations

ASO: Anti-sense oligonucleotide
CAFs: Cancer-associated fibroblasts
CLL: Chronic lymphocytic leukemia
CRISPR: Clustered Regularly Interspaced Short Palindromic Repeat
EMT: Epithelial–mesenchymal transition
ES: Exon skipping
GC: Gastric cancer
HCC: Hepatocellular carcinoma
HTS: High-throughput screening
IHC: Immunohistochemistry
IR: Intron retention
iTRAQ: Isobaric tagging for absolute quantitation
KO: Knockout
LC/MS/MS: Liquid Chromatography Tandem-Mass Spectrometry
LN: Lymph node
LOH: Loss of heterozygosity
MET: Mesenchymal epithelial transition
miRNA: microRNA or miR
ncRNA: non-coding RNA
NFs: Normal fibroblasts
PKM: Pyruvate Kinase muscle isoenzyme
PPI: Protein-protein interaction
PTM: Post-translational modifications
PSEN1: Presenilin 1
RAP1-A: Ras-related protein Rap-1A
RNAi: RNA interference
RPKM: Reads per kilo base per million mapped reads
SCID: Severe combined immunodeficiency
SIAH1: Siah E3 ubiquitin protein ligase 1
SILAC: Stable isotope labeling in animal cell culture
SMT: Somatic mutation theory
SPLMs: Splicing modulators
SOC: Standard of care
SRSF1: Serine and arginine rich splicing factor 1
SS: Splice site
STAT3: Signal transducer and activator of transcription 3
TAM: Tumor associated macrophages
TALEN: Transcription activator-like effector nucleases
TCTP1: Translationally controlled tumor protein 1
TFs: Transcription factors
TME: Tumor microenvironment
TOFT: Tissue organization field theory
TSAP6: Tumor suppressor activated pathway 6
TSG: Tumor suppressor gene
UTR: Untranslated region
Y2H: Yeast two-hybrid
ZFN: Zinc finger nuclease
